# Persistent Physical Symptoms and Psychosomatic Profiling in Fibromyalgia: A Cross-Sectional Study in a Pain Management Unit

**DOI:** 10.3390/jcm15051964

**Published:** 2026-03-04

**Authors:** Jose A. Cabero-Pérez, Julen Susperregui, Mª José Diez, Irene Solera-Ruiz, Alicia Alonso-Cardaño, Sergio Batuecas-Asensio, Antonio Serrano-García

**Affiliations:** 1 Anesthesiology, Resuscitation and Pain Management, Complejo Asistencial Universitario de Leon, 24008 Leon, Spain; isolera@saludcastillayleon.es (I.S.-R.); aalonsocar@saludcastillayleon.es (A.A.-C.); sbatuecas@saludcastillayleon.es (S.B.-A.); 2 Applied Mathematics, Department of Mathematics, University of Leon, 24007 Leon, Spain; jsusl@unileon.es; 3 Department of Biomedical Sciences, Veterinary Faculty, Institute of Biomedicine (IBIOMED), University of Leon, 24007 Leon, Spain; mjdiel@unileon.es; 4 Psychiatry Service, Department of Psychosomatic, Complejo Asistencial Universitario de Leon, 24008 Leon, Spain; aserranoga@saludcastillayleon.es

**Keywords:** fibromyalgia, persistent physical symptoms, Modified Somatic Perception Questionnaire, Zung, Distress and Risk Assessment Method

## Abstract

**Background/Objectives**: Fibromyalgia is a chronic pain syndrome influenced by both physical and psychological factors, but their interaction remains unclear. We evaluated tools combining physical and emotional dimensions to characterise fibromyalgia and assess associations with persistent physical symptoms (PPS) and the emotional distress in its clinical interpretation. **Methods**: This cross-sectional study included 1588 patients referred to the Pain Management Unit, Complejo Asistencial Universitario de León. Fibromyalgia cases had a prior diagnosis to referral using the 2019 ACTTION–American Pain Society Pain Taxonomy diagnostic criteria. At the first consultation, participants completed a standardized protocol including sociodemographic variables, a Central Sensitization Inventory Part B checklist of previously physician-diagnosed physical and psychological conditions, and the visual analogue scale for pain, the Modified Somatic Perception Questionnaire (MSPQ), and the Zung Self-Rating Depression Scale. The Distress and Risk Assessment Method (DRAM) was used to integrate MSPQ and Zung data. **Results**: Women had higher odds of fibromyalgia (*p* = 0.003). Fibromyalgia was associated with PPS (*p* < 0.001), with chronic fatigue predominating in women (*p* < 0.001) and neck injury/whiplash in men (*p* = 0.005). The MSPQ had the highest OR among the instruments evaluated (overall: *p* < 0.001; women: *p* < 0.001; men: *p* = 0.005). Fibromyalgia status differed by DRAM category (nominal model, *p* < 0.001), suggesting higher odds in the Depressive and Somatic categories compared with Normal. **Conclusions**: In our sample, sex was associated with fibromyalgia and PPS profiles; PPS profiles were also associated with fibromyalgia. The MSPQ appeared to be among the most informative instruments, and its integration with Zung through the DRAM may have potential utility for psychosomatic risk profiling in fibromyalgia, warranting further study.

## 1. Introduction

Fibromyalgia is characterised by widespread musculoskeletal pain with persistent fatigue, sleep disturbance, and cognitive and affective symptoms that impair quality of life [1]. Prevalence is 2–8% worldwide, with geographic variability and higher frequency among women aged 30–50 years [2].

Fibromyalgia is thought to have multifactorial causes, involving genetic susceptibility, and complex interactions between neurobiological, neuroendocrine and immune-related alterations, and environmental influences, particularly psychological and physical stressors, that may modulate symptom onset and maintenance [3,4]. Depression and anxiety symptoms, together with heightened somatic perception, are common and clinically important in chronic pain and fibromyalgia because they are associated with symptom burden and functional impact; however, they are not universal and should not be assumed to be present in all patients [5]. Recent literature suggests that the relationship between fibromyalgia and depression is bidirectional: depressive symptoms may precede fibromyalgia in some individuals, whereas in others they may develop alongside or following sustained symptom burden, so a single direction of causality should not be assumed [6,7]. In specialised pain settings, a broader assessment may be appropriate, considering affective and cognitive symptoms in addition to pain intensity, as cognitive complaints are frequently reported and may be clinically relevant [4]. It is important to consider that fibromyalgia can be conceptualized as one phenotype within a broader spectrum of persistent physical symptoms (PPS) or central sensitisation syndromes (CSS), which often overlap clinically and may share central pain amplification mechanisms in a substantial subset of patients [8,9,10]. The term PPS is commonly used as an umbrella construct for distressing somatic complaints that persist over time, regardless of their cause [11]. In the present study, PPS were operationalised using the diagnostic checklist included in Part B of the Central Sensitization Inventory (CSI) [12], whereas fibromyalgia was defined according to the AAPT Diagnostic Criteria for Fibromyalgia [13]. In this framework, PPS and fibromyalgia are not treated as interchangeable diagnoses; rather, fibromyalgia can be regarded as a prototypical CSS that often overlaps with other persistent symptom presentations [14]. Thus, it seems crucial to systematically explore the presence of PPS, rather than focusing attention exclusively on the diagnosis of fibromyalgia. This is consistent with a non-dualistic approach in which ‘physical’ and ‘psychological’ symptoms are interdependent [15].

Accordingly, improved characterisation and treatment planning can be supported by validated psychometric instruments to evaluate emotional distress and somatic burden [16,17]. However, instruments commonly used in fibromyalgia assessments, such as the Widespread Pain Index, the Symptom Severity Scale and the Fibromyalgia Impact Questionnaire [18], as well as screening tests such as the Fibromyalgia Rapid Screening Tool [19], do not assess anxiety and depression. Mood-state protocols such as the Zung Self-Rating Depression Scale [20] may therefore be essential. In parallel, clinicians should assess somatisation, defined as a tendency to experience and report physical symptoms in an amplified manner, which has been linked to greater pain, fatigue, and functional disability [21,22,23]. The Modified Somatic Perception Questionnaire (MSPQ) can support this evaluation in pain management units and was originally developed in chronic pain settings to quantify somatic/autonomic symptom perception [24]. Despite the clinical usefulness of these scales, existing literature on the use of the MSPQ alone or combined with Zung’s scale through the Distress and Risk Assessment Method (DRAM) [25] remains limited in fibromyalgia. DRAM combines MSPQ with a modified Zung depression index to classify patients into distress risk categories and was originally proposed in spine-related pain populations [25]. Although developed in that context, the core domains captured by DRAM (depressive symptom burden and heightened somatic symptom perception) are also clinically relevant in fibromyalgia; however, published applications of DRAM in fibromyalgia appear to be scarce and, to our knowledge, have been reported in only one study to date [26]. This gap underscores the need to clarify emotional and somatic determinants of pain in fibromyalgia to inform interdisciplinary interventions and healthcare planning [27]. In this context, the present study evaluates instruments covering both physical and emotional dimensions to better characterise fibromyalgia and examines how PPS and emotional distress may influence clinical interpretation using the CSI Part B conditions checklist to capture previously physician-diagnosed related conditions.

## 2. Materials and Methods

### 2.1. Study Population

This observational, cross-sectional study included 1588 adult patients (≥18 years) from the database of the Pain Management Unit, Department of Anesthesiology, Resuscitation and Pain Management (Complejo Asistencial Universitario de León, Spain) between January 2021 and December 2024, analysed according to fibromyalgia status (fibromyalgia versus non-fibromyalgia). The minimum required sample size was calculated to estimate a proportion with a margin of error of 0.05 and a 95% confidence level. The resulting minimum sample size was 385. For patients with fibromyalgia, the diagnosis had been established prior to referral to the Pain Management Unit, according to criteria described in an updated review [13], which superseded the original 1990 American College of Rheumatology criteria [28]. Diagnoses were accepted as documented at referral and were not re-adjudicated within the unit; therefore, some inter-clinician variability in diagnostic application cannot be fully excluded. At the first visit, patients completed the standardised pain assessment questionnaire; a healthcare professional was present to clarify item wording and assist with interpretation when needed, without guiding responses. Eligible participants had sufficient reading comprehension to complete written materials.

The study was conducted in accordance with the Declaration of Helsinki and its subsequent amendments or comparable ethical standards, and it was approved by the Ethics Committee of the Complejo Asistencial Universitario de León (CAULE, no. 25028). Additionally, the study adhered to Spain’s Organic Law 3/2018 of 5 December on the Protection of Personal Data and the Guarantee of Digital Rights, ensuring the confidentiality of participant information and its secure storage in accordance with applicable data protection requirements. The study is reported according to the Strengthening the Reporting of Observational Studies in Epidemiology (STROBE) statement [29].

### 2.2. Variables Analysed

The protocol completed by patients at their first visit included an introductory section explaining the main objective of the study. The following sociodemographic variables were then collected: sex, date of birth (to calculate age in years), height (m), weight (kg), employment status (employed, unemployed, work-disabled, temporal work-disabled, retired and homemaker), employment arrangement (salaried employed and self-employed) and educational level (Primary education or below, Secondary education and Higher education). Body mass index (BMI) was calculated from patient-reported height and weight, and the following categories were defined: underweight, <18.5 kg/m^2^; normal weight, 18.5 ≤ BMI < 25 kg/m^2^; overweight, 25 ≤ BMI < 30 kg/m^2^ and obesity, ≥30 kg/m^2^.

Another section of the survey enquired about prior physician-diagnosed conditions, using the diagnostic checklist included in Part B of the CSI [12]. Part B is a non-scored section that records conditions previously diagnosed by a healthcare professional. Accordingly, participants were instructed to tick only those conditions that had been previously diagnosed by a physician, and no new diagnoses were established in our unit. Although CSI Part B includes an optional field for the year of diagnosis, this information was not collected in our study; therefore, checklist responses were used only to indicate whether each condition had ever been diagnosed, without allowing assessment of recency, symptom duration, or current status. This checklist covered seven CSS (restless legs syndrome, fibromyalgia, chronic fatigue syndrome, temporomandibular joint disorder, migraine or tension headache, irritable bowel syndrome, multiple chemical sensitivity) and three CSS-related disorders (neck injury/whiplash, anxiety or panic attacks, and depression). In this study, PPS is used operationally to refer to CSI Part B physical CSS diagnoses and neck injury/whiplash, whereas anxiety/depression are presented separately as emotional comorbidity diagnoses. Because fibromyalgia was the index diagnosis of the study, defined separately using the AAPT criteria, the CSI Part B fibromyalgia item was recorded but not incorporated into the PPS construct/variable used in the analyses.

In the final section, patients completed the following scales/questionnaires: the Visual Analogue Scale for pain (VAS), the Modified Somatic Perception Questionnaire (MSPQ), and the Zung Self-Rating Depression Scale. In addition, we used the Distress and Risk Assessment Method (DRAM) to integrate MSPQ and Zung Self-Rating Depression Scale scores. Scores were dichotomized using prespecified, clinically meaningful and literature-supported cut-offs to distinguish clinically relevant symptom levels from lower levels. 

The VAS is a simple and widely used tool to quantify subjective pain intensity [30]. It is typically presented as a 100 mm horizontal line with verbal anchors at the extremes, such as ‘no pain’ and ‘the most severe pain imaginable’ [30]. The VAS score is obtained by measuring (in millimetres) the distance from the ‘no pain’ anchor to the patient’s mark, yielding a continuous score from 0 to 100, with higher scores indicating greater pain intensity [30]. Pain intensity on the VAS was dichotomised using a prespecified cut-off of ≥70/100 mm to indicate severe pain, consistent with published interpretability cut-points for a 100-mm VAS [31]. This threshold aligns with commonly used severity grading on 0–10 pain scales, where scores of 7–10 indicate severe pain [32]. Psychometric evaluations have reported good to excellent test-retest reliability for pain VAS scores and supporting evidence of validity in adult pain populations [30]. 

The MSPQ is commonly used to assess the perception of somatic symptoms [21]. In this study, we used the MSPQ in its standard 22-item format, in which patients respond on a 0–3 Likert-type scale, where 0 indicates ‘not at all’ and 3 corresponds to ‘extremely, cannot be worse’ [21]. In accordance with the original scoring approach, the MSPQ total score is derived from 13 of the 22 items and is calculated as the direct sum of items 2, 3, 7, 8, 9, 11, 13, 14, 16, 18, 19, 20 and 21, yielding a total score range of 0-39, with higher scores indicating greater somatic symptom perception [21]. For interpretability, we dichotomised the MSPQ using a prespecified cut-off score of 12, which was used to indicate an increased response to bodily perceptions [25]. In clinical back pain samples, the MSPQ has shown good internal consistency (Cronbach’s alpha = 0.78) and evidence of construct validity [33]. 

The Zung Self-Rating Depression Scale is a 20-item self-report questionnaire designed to assess depressive symptom severity [20]. In this study, we used the Spanish validated (Castilian) adaptation of the Zung Self-Rating Depression Scale [34]. Items are rated on a 1–4 scale assessing the proportion of time the patient experiences the situations described, where 1 indicates ‘almost never’, and 4 indicates ‘always’. Total scores range from 20 to 80 (raw total score), with higher scores indicating greater depressive symptom severity. In the Spanish version, commonly used interpretive cut-offs classify scores of 20–35 as no depression, 36–51 as mild depression, 52–67 as moderate depression, and ≥68 as severe depression [34]. For the purposes of this study, we considered Zung scores ≥52 as indicative of depressive symptomatology. Spanish-language validation studies have indicated high internal consistency for the Zung Self-Rating Depression Scale (Cronbach’s alpha = 0.89) and good ability to discriminate clinically diagnosed depression in validation settings, with ROC AUC values of approximately 0.84–0.96, depending on comparison groups [35]. Additional evidence supporting diagnostic performance in Spanish primary care samples has also been reported [36].

Given the influence that both psychological and somatic factors appear to exert on the various manifestations of fibromyalgia, we explored whether combining these two scales could help to profile patients. The Distress and Risk Assessment Method (DRAM) is a brief screening and patient profiling classification method originally developed by Main et al. to identify psychosocial distress using combined information from the MSPQ and the Zung Self-Rating Depression Scale [25]. In this study, we applied the Spanish translation of the DRAM as operationalised in a Spanish clinical setting [37]. Scoring and classification follow DRAM framework, which integrates scores from the MSPQ and the Zung Self-Rating Depression Scale and assigns patients to four categories based on prespecified cut-offs [25,37]. DRAM categories were defined using the cut-offs reported by Serrano-García et al. [37]:

- Normal (A): Zung score < 36.
- At risk (B): Zung score 36–51 and MSPQ < 12.
- Distressed-depressive (C depressive): Zung score ≥ 52.
- Distressed-somatic (C somatic): Zung score 36–51 and MSPQ ≥ 12.

Accordingly, ‘at risk’ and ‘distressed’ categories suggest higher psychosocial distress and the method has been used to support risk stratification and prediction of poorer clinical outcomes [25,37].

### 2.3. Statistical Analysis

Descriptive statistics were used to summarise the study variables, reporting frequencies (percentages) for categorical data and medians with interquartile ranges (IQRs) for quantitative data. Pearson’s chi-squared test or Fisher’s exact test, as appropriate, was used to compare categorical variables. Odds ratios (ORs) with corresponding 95% confidence intervals (CIs) were calculated. Bivariable analyses were performed to examine the association of each PPS, anxiety/depression, and questionnaire-derived measure (VAS, MSPQ, and Zung) with fibromyalgia. Stepwise multivariable binary logistic regression was then performed to estimate adjusted associations with fibromyalgia, reporting adjusted ORs with 95% CIs. Stepwise selection was used for exploratory purposes to compare the relative contribution of predictors. Multicollinearity among predictors was assessed using variance inflation factors (VIFs), with values <2 considered acceptable. Model calibration was assessed using the Hosmer–Lemeshow goodness-of-fit test. Sex-stratified analyses were conducted by repeating the multivariable models separately in women and men for selected outcomes, including persistent physical symptoms and emotional symptoms (anxiety and depression) and questionnaire scores (VAS, MSPQ, Zung, and DRAM). DRAM categories were entered in regression models as a categorical factor using indicator (dummy) variables, rather than as an equidistant ordinal predictor. Missing data occurred; analyses were conducted using available data, and no imputation procedures were applied. The final analytic sample provided a maximum half-width of the 95% CI margin of error below 0.025. All analyses were performed using IBM SPSS Statistics version 29 (IBM Corporation, Armonk, NY, USA), and statistical significance was set at *p* < 0.05.

## 3. Results

### 3.1. Sociodemographic Characteristics of the Study Population

Among the 1588 patients, 119 (7.5%) had a prior diagnosis of fibromyalgia. Of the sociodemographic variables examined, sex was significantly associated with the likelihood of fibromyalgia. Specifically, fibromyalgia was 2.367 times more likely in women than in men (OR = 2.367; 95% CI 1.340–4.181; *p* = 0.003). When these sociodemographic variables were analysed exploratorily, stratified by sex, only in women was each one-category decrease in BMI associated with higher odds of fibromyalgia (OR = 1.451; 95% CI 1.065–1.976; *p* = 0.018). None of the other characteristics analysed were associated with the likelihood of fibromyalgia. The general sociodemographic characteristics of patients with and without fibromyalgia are presented in Table 1, which also reports the extent of missing data for the variables shown.

### 3.2. Relationship of Fibromyalgia with Persistent Physical Symptoms and the Emotional Component

Patients with fibromyalgia were found to report a range of PPS, including chronic fatigue syndrome, temporomandibular joint disorder, migraine or tension headache, irritable bowel syndrome, restless legs syndrome and multiple chemical sensitivity and neck injury/whiplash; as well as an emotional component characterised by anxiety and depression. Pairwise comparisons were conducted to assess the association of each PPS and each emotional comorbidity with fibromyalgia. All PPS and emotional variables were significantly associated with fibromyalgia (*p* < 0.001). Based on these findings, we conducted a stepwise binary logistic regression to identify which PPS and emotional comorbidities exerted the greatest influence on the presence of fibromyalgia. Multicollinearity was assessed using VIFs and was not a concern (all VIFs ≤ 1.406; Appendix A.1 Table A1). The variables showing the strongest associations with fibromyalgia were chronic fatigue syndrome, irritable bowel syndrome, neck injury/whiplash, migraine or tension headache and the emotional variable depression (Table 2). When the stepwise multivariable logistic regression was repeated separately in women and men, the pattern of PPS associated with fibromyalgia differed by sex: chronic fatigue syndrome showed the strongest association in women, whereas neck injury/whiplash did so in men. In women, depression was detected as an emotional variable associated with fibromyalgia, while such an association was not observed in men (Table 3).

### 3.3. Evaluation of the VAS, MSPQ and Zung Scales in Relation to Fibromyalgia

Separate analyses showed that each scale, taking into account the established cut-off points, was significantly associated with fibromyalgia; these associations were statistically significant in bivariate chi-square tests (Table 4). Questionnaire-specific missing data (VAS, MSPQ, Zung, and DRAM) are summarised in Appendix A.2 Table A2. We also performed a stepwise logistic regression to determine the influence of each scale used in this study on the assessment of patients with a prior diagnosis of fibromyalgia. This analysis showed that MSPQ had the highest chi-squared value among the three scales, and the global test across the three dichotomised variables was significant. Patients with a positive MSPQ result (score ≥ 12) were four times more likely to have a fibromyalgia diagnosis (OR = 4.002; 95% CI 2.586–6.192; *p* < 0.001), while the relative effects of the VAS and Zung scales were not statistically significant. This does not preclude their clinical usefulness; rather, it indicates that MSPQ contributed the largest independent signal in this analysis.

When binary logistic regression models were fitted for each scale separately, reflecting a clinical scenario in which only one is administered rather than all three concurrently, as in our current practice, the MSPQ again demonstrated the best performance (Table 5). Across sexes, MSPQ positivity and Zung-defined depression were associated with higher odds of fibromyalgia, whereas severe pain (VAS ≥ 7) was associated with fibromyalgia in women but not in men (Table 6). Stepwise binary logistic regression, including the three scales jointly, while accounting for patient sex, again showed that the MSPQ showed the strongest statistical association and accounted for the overall contribution of the three scales combined. Specifically, among women, a positive MSPQ result was associated with 3.5-fold higher odds of fibromyalgia (OR = 3.530; 95% CI 2.141–5.817; *p* < 0.001), whereas among men the likelihood of a fibromyalgia diagnosis was fourfold higher (OR = 4.074; 95% CI 1.611–10.307; *p* = 0.003).

### 3.4. DRAM Analysis in Relation to Fibromyalgia

In a sensitivity analysis treating DRAM categories as nominal (Normal as the reference), the odds of fibromyalgia differed significantly across DRAM categories in the overall sample (*p* < 0.001). Compared with the Normal category, the Depressive category showed increased odds of fibromyalgia (OR 3.97, *p* = 0.002; 95% CI 1.658–9.206), and the Somatic category also showed increased odds (OR 3.512, *p* = 0.011; 95% CI 1.326–9.301), whereas the Risk category did not differ from Normal (OR 1.057, *p* = 0.906; 95% CI 0.422–2.647).

When stratified by sex, the overall association remained significant in women (*p* < 0.001), with higher odds in the Depressive category versus Normal (OR 2.924, *p* = 0.027; 95% CI 1.131–7.556). In men, the global test was significant (*p* = 0.026), but category-specific estimates versus Normal were imprecise with wide confidence intervals: Depressive OR 6.392, *p* = 0.078; Somatic OR 6.789, *p* = 0.102, consistent with limited precision in this subgroup. Complete tabulated results for the sex-stratified models are provided in the Appendix A.3 (Table A3).

## 4. Discussion

This study aimed to characterize persistent physical symptoms and related psychosocial measures in patients with fibromyalgia, to provide clinically relevant information that could help guide their assessment and management. It should be noted that, whereas most research on fibromyalgia focuses on female populations, the present study examines this syndrome in both women and men to delineate possible differences. Under this premise, our study found a significant association between fibromyalgia and the PPS and emotional symptoms, with patterns of association differing by sex. The importance of MSPQ in the evaluation of these patients was also supported by our analysis, and the DRAM was contextualised in fibromyalgia, having thus far been used in individuals undergoing spinal surgery [37]. Consistent with the co-occurrence of PPS and emotional comorbidity in our cohort, the findings of this study may be situated within contemporary fibromyalgia frameworks that integrate biopsychosocial factors with altered nociceptive processing, often discussed under the constructs of nociplastic pain and central sensitization [38,39,40]. Within this framework, the measures included in our assessment capture key psychosocial and somatic symptom dimensions that are thought to contribute to fibromyalgia symptom expression and persistence, in line with distributed central processing models such as the neuromatrix concept [41]. Because participants were recruited from a specialised pain management unit, the findings may be most applicable to similar tertiary-care settings and should be extrapolated to community or primary care populations with caution.

The prevalence of fibromyalgia in our study falls within the range commonly reported for this syndrome, which varies from 2–8% [2], although higher data have been described, owing to economic factors that hinder its appropriate management [42]. The sociodemographic analysis of the study population indicated that women had a higher likelihood of fibromyalgia, a finding consistently reported in the literature [2,43]. The remaining sociodemographic variables assessed did not show significant differences between patients with and without fibromyalgia, with the exception that, among women, an inverse association between BMI category and fibromyalgia was observed in this exploratory sex-stratified analysis. Although a similar effect has previously been noted without quantification [42], this result contrasts with those reported by other authors [44,45]. Accordingly, this observation should be interpreted cautiously and considered hypothesis-generating pending replication in independent cohorts. Taken together, these observations underscore the complexity and heterogeneity of fibromyalgia and suggest that, aside from sex, individual characteristics may not be robust predictors of disease risk [46,47].

In our study, PPS and emotional comorbidity were significantly associated with fibromyalgia, consistent with previous reports, although prior work has generally focused on specific symptoms [10,42,43]. Patients with these comorbid conditions may experience a greater overall symptom burden than those with fibromyalgia alone, which can complicate the clinical distinction between pain attributable to comorbidities and the central pain characteristic of fibromyalgia [43,48]. In the analysed population, the PPS, such as chronic fatigue syndrome, irritable bowel syndrome, neck injury/whiplash, and migraine or tension headache, together with the emotional comorbidity of depression, showed the strongest associations with a diagnosis of fibromyalgia. This is consistent with previous studies identifying chronic fatigue [49] and depression [6,50] as conditions closely related to fibromyalgia. Although sex-related differences in fibromyalgia manifestations have been described, evidence regarding sex-specific patterns of individual symptom associations remains limited and heterogeneous across studies [51,52]. In our sex-stratified model, the pattern of PPS was different between women and men, with chronic fatigue syndrome showing the strongest association in women and neck injury/whiplash in men. Notably, depression was associated with fibromyalgia in women only, which may suggest that affective symptoms may contribute differently to fibromyalgia-related symptom profiles between sexes. In particular, it has been suggested that the relationship between depressive symptoms and fibromyalgia-related features may be more prominent in women than in men [53], which is consistent with our sex-stratified models. All of this justifies conducting further studies that explicitly analyse symptom profiles by sex to confirm whether these findings are generalisable to other clinical settings. This updated knowledge should be considered potentially beneficial so that clinicians can diagnose and treat patients earlier, which in turn could benefit them, as other authors have suggested [52].

Assessing the emotional and somatic components accompanying fibromyalgia with validated questionnaires is considered a priority [54]. In this context and given that patients referred to the Pain Management Unit at our center were required to complete the VAS along with two questionnaires (MSPQ and Zung), we aimed to determine whether any of these instruments was more associated with fibromyalgia. Although each instrument individually showed a statistically significant association with this diagnosis, the MSPQ emerged as the most relevant; indeed, once its influence was removed, neither the VAS nor the Zung questionnaire remained significant. These findings suggest that, in patients with fibromyalgia, the information obtained from the MSPQ could be sufficiently indicative to potentially reduce the questionnaire burden on the patient and may facilitate clinicians’ management of patient-reported data. These results are in line with research highlighting the importance of assessing fibromyalgia from a psychosomatic perspective [46,55]. Given reported sex differences in fibromyalgia manifestations [43], we further explored whether questionnaire-derived information differed by sex and observed similar individual utility of the MSPQ in both women and men. These results suggest the clinical usefulness of the MSPQ for assessing patients with fibromyalgia, even in the presence of sex-related differences.

To estimate the impact of psychosocial factors associated with fibromyalgia [56], we applied the DRAM, which integrates scores from the MSPQ and the Zung Self-Rating Depression Scale [20,21,25]. Consistent with previous reports [25], fibromyalgia status differed across DRAM categories when it was modelled as a categorical factor. Compared with the Normal category, the Depressive and Somatic categories were associated with higher odds of being in the fibromyalgia group, whereas the Risk category did not differ clearly from Normal. When analyses were stratified by sex, the association remained statistically significant in women, while in men, the overall association was significant, but category-specific estimates were imprecise. Given that the DRAM was developed as a brief distress-screening classification based on MSPQ and Zung scores, these findings suggest its potential utility for stratifying psychosocial profiles in fibromyalgia rather than for informing management decisions on its own. Profiling psychosocial risk in patients with fibromyalgia using the DRAM may have potential clinical usefulness and, in the future, could inform more targeted psychosocial assessment and, when clinically indicated, referral or tailored interventions, consistent with integrative biopsychosocial frameworks in fibromyalgia and CSS. Nevertheless, further studies are required to examine this potential application in greater depth.

### Study Limitations

This cross-sectional study allows the identification of associations but precludes causal inference. Participants were selected from a single-centre pain management unit. Persistent physical and emotional symptoms and questionnaire outcomes were self-reported (using validated instruments), and a degree of reporting bias cannot be excluded. Item-level nonresponse in self-administered questionnaires led to missing data handled by complete-case analyses without imputation; because reasons for missingness were unknown, we could not assess whether it was random, and selection bias related to missingness cannot be ruled out. Fibromyalgia diagnoses were established prior to referral and were not re-adjudicated at our unit; therefore, some diagnostic heterogeneity related to inter-clinician variability cannot be excluded. In addition, we did not collect information on when the original diagnosis was made.

From an analytical perspective, dichotomisation of VAS/MSPQ/Zung may have reduced statistical power and masked dose–response patterns, and stepwise model selection may increase overfitting risk and yield fewer stable estimates; therefore, multivariable findings should be interpreted cautiously. In addition, other commonly used sensitisation/distress measures (CSI, PHQ-15 and PSD) were not included, which limits direct comparisons with other studies. Nevertheless, the large clinical sample and the use of validated measures provide a reasonable basis for interpreting the observed associations.

## 5. Conclusions

In this study, we observed an association between sex and fibromyalgia. Fibromyalgia was also associated with PPS and emotional comorbidities, and these variables appeared to show sex-related differences in their presentation in the Pain Management Unit sample analyzed. Among the instruments evaluated, the MSPQ appeared to be among the most informative for the assessment of patients with fibromyalgia in this clinical setting. In addition, the integration of data from this questionnaire with the Zung Self-Rating Depression Scale through the DRAM may offer potential utility in similar patients referred to comparable Pain Management Units by capturing emotional dimensions that could be clinically relevant. Taken together, our findings are consistent with an integrated biopsychosocial conceptualisation of fibromyalgia within the clinical context studied, in which persistent physical and emotional symptoms may be viewed as interrelated components of a clinical presentation in this setting, rather than being treated as separate physical and psychological domains within a dualistic framework.

## Figures and Tables

**Table 1:** Sociodemographic characteristics of the study population by fibromyalgia status.

Characteristics	Non-Fibromyalgia (*n* = 1469)	Fibromyalgia (*n* = 119)
Gender		
Women	891 (60.7)	91 (76.5)
Men	578 (39.3)	28 (23.5)
Age, years	56.7 (48.8–64.0)	59.2 (49.0–66.3)
BMI ^1^		
Underweight	24 (1.6)	3 (2.5)
Normal weight	389 (26.5)	32 (26.9)
Overweight	291 (19.8)	13 (10.9)
Obesity	424 (28.9)	29 (24.4)
Not available	341 (23.2)	42 (35.3)
Employment situation		
Employed	453 (30.8)	19 (16.0)
Unemployed	163 (11.1)	14 (11.8)
Work-disabled	198 (13.5)	24 (20.2)
Temporal work-disabled	315 (21.4)	24 (20.2)
Retired	203 (13.8)	17 (14.3)
Homemaker	59 (4.0)	6 (5.0)
Not available	78 (5.3)	15 (12.6)
Employment status		
Salaried employed	661 (45.0)	31 (26.1)
Self-employed	709 (48.3)	73 (61.3)
Not available	99 (6.7)	15 (12.6)
Education		
Primary school or below	722 (49.2)	49 (41.2)
Middle or high school	440 (30.0)	18 (15.1)
University	161 (11.0)	12 (10.1)
Not available	146 (9.9)	40 (33.6)

^1^ Body mass index. Values are *n* (%) unless otherwise stated. Age is presented as median (IQR). Percentages are calculated using the total number of patients in each group (fibromyalgia *n* = 119; non-fibromyalgia *n* = 1469). “Not available” indicates missing data.

**Table 2:** Relationship of fibromyalgia with diagnosed persistent physical symptoms and emotional comorbidities.

PPS ^1^/EC ^2^	OR ^3^	Confidence Intervals (95%)		*p*
		Lower	Upper	
Chronic fatigue syndrome	2.989	1.907	4.686	<0.001
Irritable bowel syndrome	2.290	1.474	3.558	<0.001
Neck injury/whiplash	2.150	1.397	3.307	<0.001
Depression	1.715	1.135	2.591	0.010
Migraine or tension headache	1.529	1.006	2.324	0.047

^1^ Persistent physical symptom; ^2^ emotional comorbidities; ^3^, odds ratio.

**Table 3:** Relationship of fibromyalgia with diagnosed persistent physical symptoms and emotional comorbidities in women and men.

Sex	PPS/EC	OR	Confidence Intervals (95%)		*p*
			Lower	Upper	
Woman	Chronic fatigue syndrome	3.146	1.876	5.275	<0.001
	Irritable bowel syndrome	2.418	1.474	3.968	<0.001
	Neck injury/whiplash	2.062	1.276	3.333	0.003
	Depression	1.670	1.040	2.680	0.034
Man	Neck injury/whiplash	3.604	1.459	8.906	0.005
	Chronic fatigue syndrome	2.980	1.193	7.438	0.019
	Restless legs syndrome	2.900	1.191	7.060	0.019

**Table 4:** Relationship of the VAS, MSPQ and Zung scales with fibromyalgia.

Scales	No Fibromyalgia	Fibromyalgia	Chi-Square Test	*p*
VAS ^1^	1232 (86.8%)	102 (91.9%)	6.252	0.012
MSPQ ^2^	396 (28.6%)	65 (61.9%)	43.961	<0.001
Zung	452 (32.9%)	62 (59.6%)	18.354	<0.001
Global Statistics	-	-	47.393	<0.001

^1^, Visual Analogue Scale; ^2^, Modified Somatic Perception Questionnaire. Values are presented as n (%). Percentages are calculated among available cases for each variable. *p* values are from Pearson’s chi-square test. ‘Global statistics’ refers to the overall chi-square test across the three dichotomised variables shown in the table. Severe pain was defined as VAS ≥ 7; MSPQ positivity was defined as ≥ 12; depression was defined as a Zung score ≥ 52.

**Table 5:** Influence of the VAS, MSPQ and Zung scales in the assessment of fibromyalgia.

Scales	OR	Confidence Intervals (95%)		*p*
		Lower	Upper	
VAS	2.146	1.270	3.626	0.004
MSPQ	3.711	2.478	5.558	<0.001
Zung	2.257	1.599	3.186	<0.001

Severe pain was defined as VAS ≥ 7; MSPQ positivity was defined as ≥12; depression was defined as a Zung score ≥ 52.

**Table 6:** Individual influence of the VAS, MSPQ and Zung scales on the assessment of fibromyalgia depending on sex.

Scale	Sex	OR	Confidence Intervals (95%)		*p*
			Lower	Upper	
VAS	Woman	2.284	1.196	4.362	0.012
	Man	-	-	-	>0.05
MSPQ	Woman	3.231	2.047	5.099	<0.001
	Man	3.690	1.490	9.141	0.005
Zung	Woman	2.126	1.434	3.151	<0.001
	Man	2.101	1.010	4.370	0.047

Severe pain was defined as VAS ≥ 7; MSPQ positivity was defined as ≥12; depression was defined as a Zung score ≥ 52.

## Data Availability

The data are not publicly available due to privacy and ethical restrictions. The dataset contains anonymised patient information collected for clinical purposes, and access is restricted to ensure confidentiality and compliance with data protection regulations.

## Abbreviations

The following abbreviations are used in this manuscript:

BMI	Body mass index
CSI	Central Sensitization Inventory
CSS	Central sensitisation syndromes
DRAM	Distress and Risk Assessment Method
MSPQ	Modified Somatic Perception Questionnaire
PPS	Persistent physical symptoms
VAS	Visual analogue scale
VIF	Variance inflation factors

## Appendix A

### Appendix A.1

**Table A1:** Variance Inflation Factors for Multicollinearity Diagnostics in the PPS and Emotional Symptoms Multivariable Model.

Variable	Variance Inflation Factors
Multiple chemical sensitivity	1.049
Irritable bowel syndrome	1.088
Temporomandibular joint disorder	1.110
Migraine or Tension headache	1.144
Restless legs syndrome	1.121
Neck injury/whiplash	1.137
Chronic fatigue syndrome	1.215
Depression	1.406
Anxiety	1.409

### Appendix A.2

**Table A2:** Missing data for questionnaire-derived variables by fibromyalgia status.

Variable	FM ^1^: Available	FM: Missing	Non-FM: Available	Non-FM: Missing	Total: Missing
VAS	111 (93.3)	8 (6.7)	1420 (96.7)	49 (3.3)	57 (3.6)
MSPQ	105 (88.2)	14 (11.8)	1386 (94.3)	83 (5.7)	97 (6.1)
Zung	104 (87.4)	15 (12.6)	1372 (93.4)	97 (6.6)	112 (7.1)
DRAM	103 (86.6)	16 (13.4)	1370 (93.3)	99 (6.7)	115 (7.2)

^1^, fibromyalgia. Values are *n* (%). Percentages are calculated using the total number of patients in each group (FM *n* = 119; non-FM *n* = 1469).

### Appendix A.3

**Table A3:** Sex-stratified association between DRAM categories and fibromyalgia status.

Sex	DRAM ^1^	OR ^2^	Confidence Intervals (95%)		*p*
			Lower	Upper	
Woman	Global				<0.001
	Normal vs. Risk	0.915	0.329	2.548	0.866
	Normal vs. Depressive	2.924	1.131	7.556	0.027
	Normal vs. Somatic	2.482	0.840	7.335	0.100
Man	Global				0.026
	Normal vs. Risk	1.558	0.180	13.518	0.688
	Normal vs. Depressive	6.392	0.811	50.366	0.078
	Normal vs. Somatic	6.789	0.684	67.392	0.102

^1^, Distress and Risk Assessment Method; ^2^, odds ratio.
